# Relation between the Sensory and Anthropometric Variables in the Quiet Standing Postural Control: Is the Inverted Pendulum Important for the Static Balance Control?

**DOI:** 10.1155/2015/985312

**Published:** 2015-10-11

**Authors:** Angélica C. Alonso, Luis Mochizuki, Natália Mariana Silva Luna, Sérgio Ayama, Alexandra Carolina Canonica, Júlia M. D. A. Greve

**Affiliations:** Laboratory for the Study of Movement, Department of Orthopedics and Traumatology, School of Medicine, University of São Paulo, Ovídeo Pires de Campos, 333 Cerqueira César, 05403-010 São Paulo, SP, Brazil

## Abstract

The aim of this study was to evaluate the relation between the sensory and anthropometric variables in the quiet standing. *Methods*. One hundred individuals (50 men, 50 women; 20–40 years old) participated in this study. For all participants, the body composition (fat tissue, lean mass, bone mineral content, and bone mineral density) and body mass, height, trunk-head length, lower limb length, and upper limb length were measured. The center of pressure was measured during the quiet standing posture, the eyes opened and closed with a force platform. Correlation and regression analysis were run to analyze the relation among body composition, anthropometric data, and postural sway. *Results*. The correlation analysis showed low relation between postural sway and anthropometric variables. The multiple linear regression analyses showed that the height explained 12% of the mediolateral displacement and 11% of the center of pressure area. The length of the trunk head explained 6% of displacement in the anteroposterior postural sway. During eyes closed condition, the support basis and height explained 18% of mediolateral postural sway. *Conclusion*. The postural control depends on body composition and dimension. This relation is mediated by the sensory information. The height was the anthropometric variable that most influenced the postural sway.

## 1. Introduction

During the quiet upright position, both internal and external factors affect the postural sway. To deal with any type of physical or physiological constraint or perturbation, sensory information is necessary for the postural control. The sensory information about posture and kinesthesia feeds the postural control to build a postural frame of reference. For the sagittal plane, the quiet upright posture can be modeled as an inverted pendulum [1] which sways mostly around the ankle joint. This condition reveals the ankle strategy [2]. On the other hand, for the frontal plane, the same orthostatic posture can be modeled as a double inverted pendulum, revealing the hip strategy [2]. The postural control uses the combination of both strategies to keep the postural sway inside the basis of support. The postural sway is the response to control the position of the center of mass [1]. Those postural strategies suggest that close loop control is important to control balance.

To control the inverted pendulum during standing, afferent sensory information is used to tune the gain of postural responses [3] and to deal with the body dimensions constraints. Then, sensory information and anthropometric variables are important factors for the postural control [3]. The variables of the inverted pendulum model that affects the postural control are its length, mass, and joint stiffness [1, 4]. Body mass changes the postural stability in prepubescent children and adolescents [5, 6], young adults [7–11], and old adults [12, 13].

The changes in sensory information about the postural frame of reference are potentially dangerous for postural control. The postural sway parameters increase during standing when the eyes are closed [14]. Chiari et al. [14] have shown that biomechanical factors (body size and body composition) are strongly related to postural sway under eyes closed condition. Therefore, the inverted pendulum model can explain what happens during standing. How important are the mechanical properties of the body for the postural control when no visual information is available? Is the inverted pendulum model able to explain how the postural control regulates the body sway?

In order to answer those questions, the aim of this study was to evaluate the relation between the sensory and anthropometric variables in the quiet standing postural control. The hypothesis of this study is that sensory information constrains the importance of the inverted pendulum variables for the postural control.

## 2. Method

### 2.1. Participants

Fifty young men and fifty young women were the participants. Their characteristics are described in Table 1. They gave their written informed consent to participate in this study and this protocol was approved by the Local Ethical Committee (number 1256/06). The inclusion criteria were as follows: no history of injury or lower limbs and trunk surgery; to be irregularly physically active over the last six months according to the International Physical Activity Questionnaire; no disease or functional impairment of the sensory system; and no current use of medications that might alter postural control. The exclusion criteria were inability to carry out the balance tests.

### 2.2. Procedures

The anthropometric and body composition variables were measured. Those measurements were always performed by the same person, who was trained previously to make such measurements. The anthropometric measurements were made according to the ISAK (International Society for the Advancement of Kinanthropometry) standards [15]. The participants' height, weight, body mass index (BMI), trunk-encephalic length, and the waist-hip ratio were recorded. The bone densitometry (LUNAR-DPX, Madison, USA), using dual energy X-ray absorptiometry (DEXA), was used to measure body composition: the percentage of fat, bone, and body lean mass.

A portable force platform (AccuSway Plus, AMTI, MA, USA) was applied to measure the ground reaction forces and moments of force during the quiet standing posture task. The sampling frequency of the forces (*F*) and moments of forces (*M*) was 100 Hz. The center of pressure was calculated according to the following equations:

(1) $$\begin{array}{c} \text{CO}{\text{P}}_{\text{AP}}=\frac{M_{\text{AP}}}{F_{\text{v}}}\\ \text{CO}{\text{P}}_{\text{ML}}=\frac{M_{\text{ML}}}{F_{\text{v}}}, \end{array}$$

where the indexes of center of pressure (COP), *M*, and *F* indicate the anteroposterior (AP), mediolateral (ML), and vertical (v) directions. The raw COP signal was filtered (10 Hz low-pass 4th order Butterworth filter) and demeaned to eliminate the foot position bias.

The participants should stand as quiet as possible on the force platform with their arms alongside the body with their eyes opened or closed for 60 seconds. Every participant repeated the task three times for each vision condition. Their feet position was marked with a template drawn on the force plate surface.

The COP variables are the root mean square (RMS) of the COP for the AP and ML directions, mean velocity, and its sway area (COP area).

### 2.3. Statistical Analysis

To check the data distribution, the Kolmogorov-Smirnov test was run. The logarithmic transformation was applied when any variable was not normally distributed. Two analyses were applied on the postural sway (dependent) and body composition and anthropometrical (independent) variables: the analysis of correlation and the multiple linear regression model (MLRM) analysis.

The MLRM analysis was applied when the variables presented *P* ≤ 0.20 in the correlation analysis. Those variables were ranked from lowest to highest *P* value. Then, the MLRM using stepwise forward selection was run and the variables were added to model one by one, according to their ranking. The variables for which *P* ≤ 0.05 were kept in the model.

## 3. Results

The anthropometric and posturographic data of the participants are described in Table 1.

### 3.1. Correlation Analysis

The correlation coefficients of the postural sway and the anthropometric and body composition variables according to visual conditions are presented in Tables 2 and 3. For the opened eyes condition, the height, waist-hip ration, trunk-cephalic length, and bone mineral composition were correlated to AP and ML COP RMS and COP area. For the closed eyes condition, the height was correlated to AP and ML COP RMS and COP area.

### 3.2. Regression Analysis

The MLRM analysis with the postural sway and the anthropometric and body composition variables for the visual conditions is described in Table 4. For the opened eyes condition, the height explained 12% of the ML COP RMS, 10% of the sway velocity, and 11% of the COP area; and the trunk-cephalic length explained 6% of the AP COP RMS. For the closed eyes condition, the height explained 18% of the ML COP RMS; and the trunk-cephalic length explained 10% of the sway velocity and 5% of the COP area.

## 4. Discussion

The aim of this study was to analyze the influence of sensory and anthropometric variables in the postural sway. The main result suggests that the visual information changes the relation of the anthropometric variables and the postural sway. When the eyes were closed, only the mediolateral postural sway could be explained by body size. This result supports the hypothesis that sensory information constrains the importance of the inverted pendulum variables for the postural sway.

Modeling the standing posture as an inverted pendulum is a strategy to reduce the number of biomechanical variables that could affect the body (center of mass) or postural (center of pressure) sways. The parameters of the inverted pendulum model are body stiffness and the inertia about the ankle [1]. Less than 20% of the postural sway (anteroposterior or mediolateral directions) could be explained by any anthropometric parameter. It suggests that the physical parameters of the body (size, mass distribution, and inertial properties of the body segments) can partly explain the behavior of the postural sway. Nevertheless, the assumption that postural sway should be normalized [14] by any body dimension must be carefully adopted.

The correlation between postural sway and body size and mass distribution was more common when the eyes were opened. The postural sway changes without the visual information [16, 17]. In general, the postural sway increases when the eyes are closed [14]. When the eyes are closed, the difference between the position of the center of mass and center of pressure increases [2] and the muscle activation at the ankle also must increase [3]. Therefore, probably the stiffness at the ankle and other joints may increase in an attempt to decrease the chance of falling.

The postural sway also reflects the ankle and hip strategies to maintain the standing position [2]. Considering the inverted pendulum model [1], anterior-posterior postural sway is related to the ankle strategy [2] and the mediolateral is related to the hip strategy when the feet are parallel [2]. Adopting this model to explain the postural sway, the motion of a pendulum depends on its length, mass, and stiffness. For the postural control, it means that the ankle strategy is affected by the body mass, height, and ankle stiffness. Our results show that trunk-cephalic length explains a small portion (6%) of the anterior-posterior postural sway and height and postural sway are positively correlated. On the other hand, the taller the participant is, the worse the balance will be [14, 18–20]. Berger et al. [21] stated that ankle displacements and the response of the gastrocnemius muscle increase with taller subjects. Allard et al. [22] and Lee and Lin [23] reported that ectomorph individuals presented greater postural sway than endomorph and mesomorph individuals because they have a higher center of mass.

The trunk-cephalic length, or the head-trunk length, was positively correlated to the mediolateral postural sway. For the hip strategy, under the inverted pendulum model, the mediolateral postural sway depends on the body mass, the head-trunk length, and the hip and lower back joints stiffness [2]. According to the regression analysis, the importance of the trunk-cephalic length for the postural sway decreased when the participants closed their eyes; otherwise, the height has increased its importance for mediolateral sway postural with closed eyes.

The waist-hip ratio was positively related to the mediolateral postural sway. Menegoni et al. [10] suggested that the waist-hip ratio leads to bad postural control. It is possible that the fat mass concentration in the chest and abdomen (android shape) increases the load on the hips, explaining the larger ML COP. Therefore, we showed that the lean mass was positively correlated to the postural control. Those results suggest that lower lean body mass and higher waist-hip ration can be risk factors for the postural control.

The absence of visual information changes the importance of body composition and dimensions. The regression analysis showed that, under the closed eyes condition, only the anthropometric variables explained the postural sway. When the visual information is suppressed, a greater importance is required from the somatosensory and vestibular systems for the postural control. The afferent information is important to set the muscle activity and tonus in an adequate level. And we just showed that the body lean mass is related to postural sway. Winters and Snow [18] correlated the bone mineral density with the anthropometric variables and found an interrelation between them; but they reported that it did not influence the postural balance.

How important are the mechanical properties of the body for the postural control when no visual information is available? If the visual information is absent, the influence of body composition on the balance postural vanishes, while the importance of the body dimensions increases. Is the inverted pendulum model able to explain how the postural control regulates the body sway? Our results suggest that the ankle and hip strategies have opposite behaviors in relation to vision and the inverted pendulum. Nevertheless, the lengths of the single and the double inverted pendulum are important for the postural sway. Attempts are proposed to understand how the nervous system controls the postural sway during standing [1, 2]. If the postural control fails during an unstable condition, the person may trip when it walks over an obstacle, falls down, and may have an injury. The higher postural sway is related to higher risk to fall down in the elderly [24].

## 5. Conclusion

The postural control depends on body composition and dimension. This relation is mediated by the sensory information. The height was the anthropometric variable that most influenced the postural sway.

## Figures and Tables

**Table 1:** Characteristics of the study population (anthropometric and posturographic).

Variables	General group mean (SD) *N* = 100
Anthropometrics	
Height (cm)	168.8 (9.5)
Body mass (kg)	69.9 (14.3)
BMI (kg/m^2^)	24.3 (3.6)
Trunk-cephalic length (cm)	89.9 (4.4)
% fat	30.2 (10.1)
Lean mass (kg)	46.9 (11.8)
Bone mineral composition (kg)	2.77 (0.55)
Waist-hip ratio (cm)	81.7 (7.6)
Posturographic measurements (log_10_⁡)	
Eyes open	
ML COP RMS (cm)	−0.685 (0.154)
AP COP RMS (cm)	−0.421 (0.128)
Sway velocity (cm/s)	−0.130 (0.097)
Displacement area (cm^2^)	0.140 (0.243)
Eyes closed	
ML COP RMS (cm)	−0.612 (0.161)
AP COP RMS (cm)	−0.332 (0.148)
Sway velocity (cm/s)	0.008 (0.110)
COP area (cm^2^)	0.306 (0.259)

ML COP RMS: medial lateral center of pressure root mean square; AP COP RMS: anteroposterior center of pressure root mean square; BMI: body mass index; SD: standard deviation.

**Table 2:** Correlation between balance and the anthropometric variables in the group, with eyes open.

Variables	ML COP RMS (cm) r (P)	AP COP RMS (cm) r (P)	Sway velocity (cm/s) r (P)	COP area r (P)
Height (cm)	0.40 (0.004)^*^	0.28 (0.005)^*^	0.33 (0.001)^*^	0.35 (*P* ≤ 0.001)^*^
Mass (kg)	0.23 (0.09)	0.24 (0.01)^*^	0.15 (0.11)	0.23 (0.02)^*^
BMI (kg/m^2^)	0.03 (0.79)	0.12 (21)	−0.05 (0.57)	0.06 (0.54)
Trunk-cephalic length (cm)	0.18 (0.20)	0.27 (0.006)^*^	0.24 (0.01)^*^	0.24 (0.01)^*^
% fat	0.03 (0.82)	−0.03 (0.73)	−0.26 (0.009)^*^	−0.14 (0.15)
Lean mass (g)	0.37 (0.007)^*^	0.21 (0.03)^*^	0.28 (0.004)^*^	0.27 (0.006)^*^
Bone mineral composition (g)	0.29 (0.03)^*^	0.22 (0.02)^*^	0.19 (0.05)^*^	0.24 (0.01)^*^
Waist-hip ratio (cm)	0.12 (0.39)	0.07 (0.43)	0.18 (0.06)	0.17 (0.08)

Pearson's coefficient (r); ^*^P ≤ 0.05, ML COP RMS: medial lateral center of pressure root mean square; AP COP RMS: anteroposterior center of pressure root mean square; BMI: body mass index.

**Table 3:** Correlation between balance and the anthropometric variables in the whole group, with eyes closed.

Variables	ML COP RMS (cm) *r* (*P*)	AP COP RMS (cm) *r* (*P*)	Sway velocity (cm/s) *r* (*P*)	COP area *r* (*P*)
Height (cm)	0.35 (*P* ≤ 0.001)^*^	0.05 (0.56)	0.31 (0.001)^*^	0.25 (0.01)^*^
Mass (kg)	0.22 (0.02)^*^	0.08 (0.42)	0.17 (0.08)	0.18 (0.07)
BMI (kg/m^2^)	0.04 (0.69)	0.06 (0.53)	−0.02 (0.84)	0.06 (0.50)
Trunk-cephalic length (cm)	0.21 (0.03)^*^	0.05 (0.60)	0.16 (0.09)	0.16 (0.10)
% fat	−0.12 (0.22)	−0.002 (0.98)	−0.18 (0.06)	−0.05 (0.59)
Lean mass (g)	0.24 (0.01)^*^	0.06 (0.54)	0.25 (0.01)^*^	0.17 (0.08)
Bone mineral composition (g)	0.22 (0.02)^*^	−0.002 (0.98)	0.16 (0.09)	0.13 (0.17)
Waist-hip ratio (cm)	0.25 (0.01)^*^	0.02 (0.81)	0.18 (0.06)	0.16 (0.09)

Pearson's coefficient (*r*); ^*^ *P* ≤ 0.05, ML COP RMS: medial lateral center of pressure root mean square; AP COP RMS: anteroposterior center of pressure root mean square; BMI: body mass index.

**Table 4:** Linear regression analysis on postural balance and the anthropometric variables for the whole group, with eyes opened and eyes closed.

Group condition	Variables	Height (cm)	Trunk-cephalic length (cm)	Lean mass (kg)	Waist-hip ratio (cm)	*r* ^2^ adjusted
		*β* (*P*)	*β* (*P*)	*β* (*P*)	*β* (*P*)	
Eyes opened	ML COP RMS (cm)	+0.006 (<0.001)	—	—	—	0.12
	AP COP RMS (cm)		+0.008 (0.006)	—	—	0.06
	Sway velocity (cm/s)	+0.003 (0.001)	—			0.10
	COP area (cm^2^)	+0.009 (<0.001)	—	—	—	0.11

Eyes closed	ML COP RMS (cm)	+0.007 (<0.001)	—	—	—	0.18
	AP COP RMS (cm)	—	—	—	—	
	Sway velocity (cm/s)	—	+0.004 (<0.001)	—	—	0.10
	COP area (cm^2^)	—	+0.007 (0.01)	—	—	0.05

*r* ^2^: *r* adjusted; ^∗^ *P* ≤ 0.05, *β*: beta value; ML COP RMS: medial lateral center of pressure root mean square; AP COP RMS: anteroposterior center of pressure root mean square.
